# Dissemination and implementation of recommendations on hypertension: the Canadian experience

**DOI:** 10.1186/1710-1492-6-S4-A10

**Published:** 2010-12-10

**Authors:** Denis Drouin

**Affiliations:** 1 Département de médecine, Pavillon Ferdinand-Vandry, Université Laval, Québec, Québec, G1V 0A6, Canada

## 

The Canadian Hypertension Education Program (CHEP) started an ambitious dissemination and implementation program (D&I) in 1999 [1]. Recent data show that control of hypertension in Canada has recently improved dramatically from 13% in 1992 [2] to 66% in 2008 [3-5]. Improved hypertension control from survey data is supported by and consistent with the data of declining Canadian standardized yearly mortality and hospitalization rates for the complications of hypertension - stroke, heart failure and acute myocardial infarction [5,6]. This achievement makes Canada a world leader in treatment and control of hypertension.

CHEP’s D&I program includes three components: dissemination, implementation and addressing barriers. Dissemination has been achieved through a passive-to-active dissemination process by publishing in multiple formats - peer-reviewed and non peer-reviewed - with content tailored to end users, including patients and their families. Another important aspect has been the development of tools to help professionals in daily decision making for the management of hypertension [1].

Implementation happens when the information is used locally and barriers to the translation of such information are addressed. Characteristics of D&I programs usually include the following elements: multifaceted, multiple audiences, multimedia, consistent information and messages; sustainable, credible, using the appropriate language, and realistic and applicable. Each barrier is specific and should be addressed individually. A barrier can be, for example, related to access to professional services, diagnostic procedures, specific therapeutic procedures or different provincial/local regulations. Some barriers may be local or systemic (for example salt/sodium added in processed food) or absence of structured care in the management of chronic diseases.

In our experience, critical success factors for guidelines implementation are: a strong methodology for the development of high quality recommendations, an annual review of the scientific literature, and endorsement and participation of leading experts and key opinion leaders.

Items listed on Table 1 can probably explain CHEP’s success.

**Table 1:** 

Branding of CHEP
An Implementation Task Force
A 5-year Business Plan
An endowed chair for “Knowledge Translation on Hypertension” to spearhead, coordinate, facilitate and maintain the process
A yearly revision/dissemination process
An Outcomes Research Task Force.
